# Relation between red blood cell distribution width and 30-day in-hospital mortality of patients with ventilator-associated pneumonia

**DOI:** 10.1186/s12879-023-08692-0

**Published:** 2023-10-18

**Authors:** Zhonghua Li, Liping Yang, Qin Xu, Feifei Wu

**Affiliations:** Department of Infection Management, Jiangsu Province, Taicang First People’s Hospital, 58 South Changsheng Road, Taicang 215400, Taicang, No P. R. China

**Keywords:** VAP, RDW, 30-day in-hospital mortality, Cohort study, Risk factor

## Abstract

**Background:**

Epidemiological studies have demonstrated an association between red blood cell distribution width (RDW) and the prognosis of pneumonia-associated diseases. However, prognostic value of RDW in patients with ventilator-associated pneumonia (VAP) has yet to be investigated. This study aimed to explore the association between RDW and in-hospital mortality in VAP patients and explore predictive value of RDW for VAP patients.

**Methods:**

This retrospective cohort study included 1,543 VAP patients from the Medical Information Mart for Intensive Care IV database 2008-2019. The primary outcome was considered to 30-day in-hospital mortality of VAP patients in this study. Non-high RDW level group was defined as <15 %, and high RDW level group as ≥15%. The possible confounding factors were screened by least absolute shrinkage and selection operator regression. Univariate and multivariate COX regression analyses were used for the assessment on the association of RDW and 30-day in-hospital mortality in VAP patients. We also performed subgroup analyses. Furthermore, a comparative analysis of RDW and sequential organ failure assessment (SOFA) score and simplified acute physiology score II (SAPS II) were performed by receiver operating characteristic (ROC) curves.

**Results:**

The 30-day in-hospital mortality of VAP patients was approximately 19.05%. After adjusting all confounding factors, high RDW was associated with 30-day in-hospital mortality among VAP patients by using non-high RDW as the reference [hazard ratio (HR) =1.29, 95% confidence interval (CI): 1.01-1.63]. Additionally, the relationship was also robust in several populations, such as patients were younger than 60 years, or had not a history of congestive heart failure, or had a history of sepsis, or had not received renal replacement therapy, or had a duration of mechanical ventilation for more than 7 days. The result of ROC indicated that RDW had a better prognostic value in predicting 30-day in-hospital mortality for VAP patients than SOFA score and SAPS II score.

**Conclusion:**

High RDW level is associated with an increased 30-day in-hospital mortality. The RDW is a promising biomarker in predicting 30-day in-hospital mortality for patients admitted to the ICU, regardless of VAP.

**Supplementary Information:**

The online version contains supplementary material available at 10.1186/s12879-023-08692-0.

## Background

Ventilator-associated pneumonia (VAP) is a prevalent nosocomial infection in the intensive care unit (ICU), occurring in patients who have undergone continuous mechanical ventilation for more than 48 hours [1]. The mortality rate attributed to ventilator-associated pneumonia (VAP) is estimated at 13% [2], with patients diagnosed with VAP having a twofold higher mortality rate compared to those without a diagnosis of VAP [3]. VAP has the consequence of lengthening the patient’s hospital stays, increasing medical expenses and mortality risk [4]. Therefore, it is crucial to focus on the mortality risk of VAP patients for assessing disease severity and implement appropriate treatment measures.

Red blood cell distribution width (RDW) is a hematological parameter that reflects variability of circulating red blood cells (RBC) size and has traditionally been utilized as a diagnostic index for anemia [5, 6]. Recently, new evidence suggests that RDW is associated with the prognosis of various diseases [7, 8]. Increased RDW indicates a severe dysregulation in erythrocyte homeostasis, which may be attributed to inflammation and oxidative stress [9]. In the study of Deniz M, et al., they pointed out that RDW levels were higher in patients who died in the ICU than in those who survived, and RDW has a good prognostic value in predicting the prognosis and mortality in patients admitted to the ICU [10]. In addition, a retrospective case study indicated that higher RDW was related to short-term adverse outcomes in community-acquired pneumonia patients [11]. However, to the best of our knowledge, prognostic value of RDW in patients with VAP has not been investigated so far.

Herein, we designed a retrospective cohort study with the aim of evaluating the association between RDW and 30-day in-hospital mortality in VAP patient, as well as exploring the predictive value of RDW for these patients.

## Methods

### Study participants

This retrospective cohort study utilized data extracted from the Medical Information Mart for Intensive Care IV (MIMIC-IV) database from 2008 to 2019. The MIMIC-IV database contains the medical records of all patients who were admitted to the ICU at Beth Israel Deaconess Medical Center from 2008 to 2019 [12]. Because all data used in this study were obtained from publicly available database, this study did not require an approval of Taicang First People’s Hospital ethics committee.

**Fig. 1:**
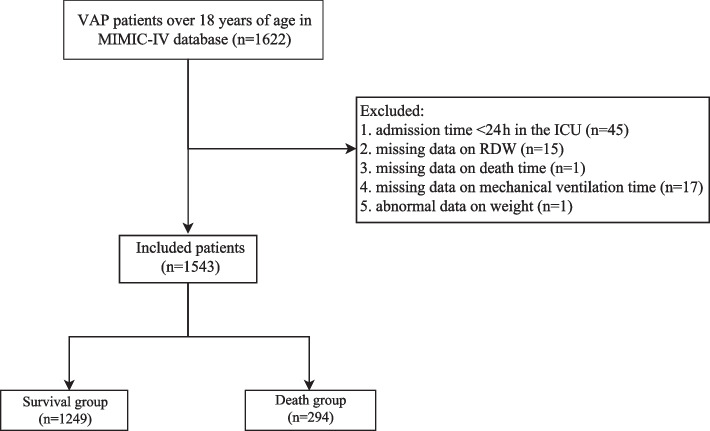
The selection process of participants

Patients who met the diagnostic criteria of VAP and were older than 18 years were included in the study. The diagnostic criteria for VAP in the MIMIC-IV database were considered as International Classification of Diseases (ICD)-9 codes of 4957, 99731 and ICD-10 codes of J95851. The exclusion criteria of participants as follows: (1) admission time <24 h in the ICU; (2) missing data on RDW or death time or mechanical ventilation time; (3) abnormal data on weight (weight=0.99kg or weight=299kg). A total of 1,543 patients diagnosed with VAP are eventually included in this study. The selection process of participants was shown in Fig. 1. All methods were performed in accordance with the Declaration of Helsinki [13].

### Outcome

In this study, patients who died within 30 days of hospital admission were considered as primary outcome event. Maximum follow-up time was 30.00 (30.00, 30.00) days.

### RDW assessment

RDW was obtained from the first record within 24h after admission to the ICU in the present study. The normal RDW value was 10%-15% [9]. In our study, non-high RDW group was defined as <15%, and high RDW group as ≥15%.

### Data collection

We extracted some data of participants from the MIMIC-IV database. Demographic information: age (years), gender, weight (kg), ethnicity, marital status, and insurance. The vital signs and laboratory data from the first record within 24h after admission to the ICU: systolic blood pressure (SBP, mmHg), diastolic blood pressure (DBP, mmHg), heart rate (bpm), respiratory rate (bpm), temperature (°C), white blood cell count (WBC, K/uL), platelet count (PLT, K/uL), hemoglobin (g/dL), RDW (%), creatinine (mg/dL), international normalized ratio (INR), blood urea nitrogen (BUN, mg/dL), glucose (mg/dL), pulse oxygen saturation (SpO2, %), and anion gap (mEq/L). Comorbidities: congestive heart failure, sepsis, and respiratory failure. Severity score: Charlson comorbidity index (CCI), Glasgow Coma Scale (GCS) score, sequential organ failure assessment (SOFA) score and simplified acute physiology score II (SAPS II). Therapy: vasopressor, renal replacement therapy (RRT), thiamine, antibiotics use, pathogen (Gram-negative bacteria, Gram-positive bacteria, and Fungus), and duration of mechanical ventilation (hour).

### Statistical analysis

Normally distributed continuous variables were expressed as Mean ± standard deviation (SD), and t-test was used to compare between two groups. Non-normally distributed continuous variables were described as median and quartiles [M (Q1, Q3)], Mann-Whitney U rank sum test was used for comparison between two groups. Categorical variables were expressed as number of cases and composition ratio [n (%)], and they were compared by adopting chi-square test. In this study, the missing values were interpolated using interpolation method, and sensitivity analysis was performed on the data before and after interpolation (Supplemental Table 1). SAS 9.4 software (SAS Institute Inc., Cary, NC, USA) and R 4.2.1 software performed all analysis.

In the present study, we used least absolute shrinkage and selection operator (LASSO) regression to screen the possible confounding factors. Univariate and multivariate COX regression analyses were used for the assessment on the association of RDW and 30-day in-hospital mortality of VAP patients. We constructed two models: Model 1 did not adjust any variables; Model 2 adjusted for age, BUN, vasopressor, RRT and duration of mechanical ventilation. Additionally, we also performed subgroup analyses based on age, congestive heart failure, sepsis, respiratory failure, RRT and duration of mechanical ventilation. Hazard ratio (HR) and 95% confidence interval (CI) was calculated. Furthermore, a comparative analysis of RDW and sequential organ failure assessment (SOFA) score and simplified acute physiology score II (SAPS II) were performed by receiver operating characteristic (ROC) curves. *P*<0.05 was deemed to possess statistical significance.

## Results

### Baseline characteristics

The study ultimately included 1,543 VAP patients, with 1,249 in the survival group and 294 in the death group. The baseline characteristic of 1,543 patients is displayed in Table 1. Furthermore, we also compared the differences in characteristics between survival group and death group. Patients in the death group were older than those in the survival group (68.39 ± 13.27 *vs* 60.01 ± 16.40, *P*<0.001). We also found that the survival group had lower RDW level than death group (15.06 ± 2.29 *vs* 15.59 ± 2.46, *P*<0.001). Compared with the survival group, VAP patients in the death group had higher creatinine, INR, CCI score, SOFA score and SAPSII score. Please see Table 1 for details.

**Table 1:** Baseline characteristics of patients with VAP

Variables	Total (*n*=1543)	Survival group (*n*=1249)	Death group (*n*=294)	*P*
Age, year, Mean ± SD	61.61 ± 16.19	60.01 ± 16.40	68.39 ± 13.27	<0.001
Gender, n (%)				0.690
Female	567 (36.75)	456 (36.51)	111 (37.76)	
Male	976 (63.25)	793 (63.49)	183 (62.24)	
Ethnicity, n (%)				0.024
White	892 (57.81)	733 (58.69)	159 (54.08)	
Black	168 (10.89)	143 (11.45)	25 (8.50)	
Other	177 (11.47)	143 (11.45)	34 (11.56)	
Unknown	306 (19.83)	230 (18.41)	76 (25.85)	
Insurance, n (%)				0.019
Medicaid	143 (9.27)	120 (9.61)	23 (7.82)	
Medicare	664 (43.03)	516 (41.31)	148 (50.34)	
Other	736 (47.70)	613 (49.08)	123 (41.84)	
Marital status, n (%)				<0.001
Divorced	123 (7.97)	88 (7.05)	35 (11.90)	
Married	758 (49.13)	604 (48.36)	154 (52.38)	
Single	505 (32.73)	437 (34.99)	68 (23.13)	
Widowed	157 (10.17)	120 (9.61)	37 (12.59)	
Weight, kg, Mean ± SD	85.16 ± 26.67	85.69 ± 27.11	82.92 ± 24.62	0.089
Heart rate, bpm, Mean ± SD	90.71 ± 21.48	90.82 ± 21.40	90.23 ± 21.86	0.677
SBP, mmHg, Mean ± SD	124.70 ± 26.60	125.02 ± 26.41	123.35 ± 27.41	0.332
DBP, mmHg, Mean ± SD	69.08 ± 18.49	69.61 ± 18.49	66.81 ± 18.32	0.019
Respiratory rate, bpm, Mean ± SD	19.88 ± 6.12	19.78 ± 6.14	20.28 ± 6.02	0.212
Temperature, ℃, Mean ± SD	36.74 ± 1.01	36.77 ± 0.99	36.58 ± 1.07	0.004
WBC, K/uL, M (Q_1_, Q_3_)	11.90 (8.70, 16.30)	11.90 (8.70, 16.10)	12.20 (9.10, 17.50)	0.155
PLT, K/uL, M (Q_1_, Q_3_)	193.00 (135.00, 254.00)	194.00 (135.00, 254.00)	187.50 (135.00, 257.00)	0.989
Hemoglobin, g/dL, Mean ± SD	10.90 ± 2.43	10.98 ± 2.43	10.57 ± 2.42	0.010
RDW, %, Mean ± SD	15.16 ± 2.33	15.06 ± 2.29	15.59 ± 2.46	<0.001
Creatinine, mg/dL, M (Q_1_, Q_3_)	1.00 (0.80, 1.60)	1.00 (0.70, 1.60)	1.30 (0.80, 1.90)	<0.001
INR, ratio, M (Q_1_, Q_3_)	1.20 (1.10, 1.50)	1.20 (1.10, 1.50)	1.30 (1.10, 1.70)	0.008
BUN, mg/dL, M (Q_1_, Q_3_)	21.00 (14.00, 34.00)	20.00 (13.00, 31.00)	26.00 (17.00, 45.00)	<0.001
Glucose, mg/dL, M (Q_1_, Q_3_)	139.00 (111.00, 181.00)	139.00 (111.00, 181.00)	138.50 (112.00, 187.00)	0.535
SPO2, %, Mean ± SD	96.92 ± 4.56	96.97 ± 4.57	96.68 ± 4.49	0.314
Anion gap, mEq/L, Mean ± SD	15.43 ± 4.82	15.25 ± 4.76	16.20 ± 5.01	0.002
Congestive heart failure (Yes), n (%)	502 (32.53)	374 (29.94)	128 (43.54)	<0.001
Sepsis (Yes), n (%)	1248 (80.88)	1003 (80.30)	245 (83.33)	0.235
Respiratory failure (Yes), n (%)	851 (55.15)	664 (53.16)	187 (63.61)	0.001
GCS, score, M (Q_1_, Q_3_)	8.00 (4.00, 12.00)	8.00 (5.00, 12.00)	7.00 (3.00, 11.00)	<0.001
CCI, score, M (Q_1_, Q_3_)	3.00 (1.00, 4.00)	2.00 (1.00, 4.00)	4.00 (2.00, 5.00)	<0.001
SOFA, score, M (Q_1_, Q_3_)	8.00 (5.00, 11.00)	8.00 (5.00, 11.00)	9.00 (6.00, 12.00)	<0.001
SAPSII, score, M (Q_1_, Q_3_)	40.00 (31.00, 50.00)	38.00 (30.00, 48.00)	46.00 (37.00, 56.00)	<0.001
Vasopressors (Yes), n (%)	1044 (67.66)	818 (65.49)	226 (76.87)	<0.001
RRT (Yes), n (%)	293 (18.99)	210 (16.81)	83 (28.23)	<0.001
Thiamine (Yes), n (%)	254 (16.46)	214 (17.13)	40 (13.61)	0.142
Antibiotics use				
Vancomycin (Yes), n (%)	1382 (89.57)	1114 (89.19)	268 (91.16)	0.321
Cephalosporins (Yes), n (%)	1228 (79.59)	994 (79.58)	234 (79.59)	0.998
Penicillin (Yes), n (%)	721 (46.73)	598 (47.88)	123 (41.84)	0.062
Aminoglycoside (Yes), n (%)	207 (13.42)	174 (13.93)	33 (11.22)	0.221
Others, n (%)	1055 (68.37)	849 (67.97)	206 (70.07)	0.487
Pathogen				
Gram-negative bacteria				
*Escherichia* *coli* (Yes), n (%)	185 (11.99)	154 (12.33)	31 (10.54)	0.396
*Klebsiella* *pneumoniae* (Yes), n (%)	172 (11.15)	138 (11.05)	34 (11.56)	0.800
*Pseudomonas* *aeruginosa* (Yes), n (%)	224 (14.52)	188 (15.05)	36 (12.24)	0.219
*Serratia* *marcescens* (Yes), n (%)	61 (3.95)	49 (3.92)	12 (4.08)	0.900
*Proteus* (Yes), n (%)	50 (3.24)	42 (3.36)	8 (2.72)	0.576
*Acinetobacter* (Yes), n (%)	53 (3.43)	38 (3.04)	15 (5.10)	0.081
*Haemophilus* *influenzae* (Yes), n (%)	115 (7.45)	97 (7.77)	18 (6.12)	0.334
Gram-positive bacteria				
*Methicillin* *resistant* *staph* *aureus* (Yes), n (%)	102 (6.61)	78 (6.24)	24 (8.16)	0.234
*Staph* *aureus* *coag* + (Yes), n (%)	477 (30.91)	389 (31.14)	88 (29.93)	0.686
*Streptococcus* *pneumoniae* (Yes), n (%)	37 (2.40)	30 (2.40)	7 (2.38)	0.983
Fungus (Yes), n (%)	133 (8.62)	101 (8.09)	32 (10.88)	0.124
Duration of mechanical ventilation, hour, M (Q_1_, Q_3_)	151.53 (76.00, 268.27)	153.00 (78.00, 278.50)	146.38 (67.18, 233.50)	0.014
Follow-up, day, M (Q_1_, Q_3_)	30.00 (30.00, 30.00)	30.00 (30.00, 30.00)	13.27 (9.14, 18.55)	<0.001

*VAP* ventilator-associated pneumonia, *SBP* systolic blood pressure, *DBP* diastolic blood pressure, *WBC* white blood cell count, *PLT* platelet count, *RDW* red blood cell distribution width, *INR* international normalized ratio, *BUN* blood urea nitrogen, *SpO2* pulse oxygen saturation, *CCI* Charlson comorbidity index, *GCS* Glasgow Coma Scale, *SOFA* sequential organ failure assessment, *SAPS II* simplified acute physiology score II, *RRT* renal replacement therapy, *SD* standard deviation.

### Association of RDW and 30-day in-hospital mortality in VAP patients

**Fig. 2:**
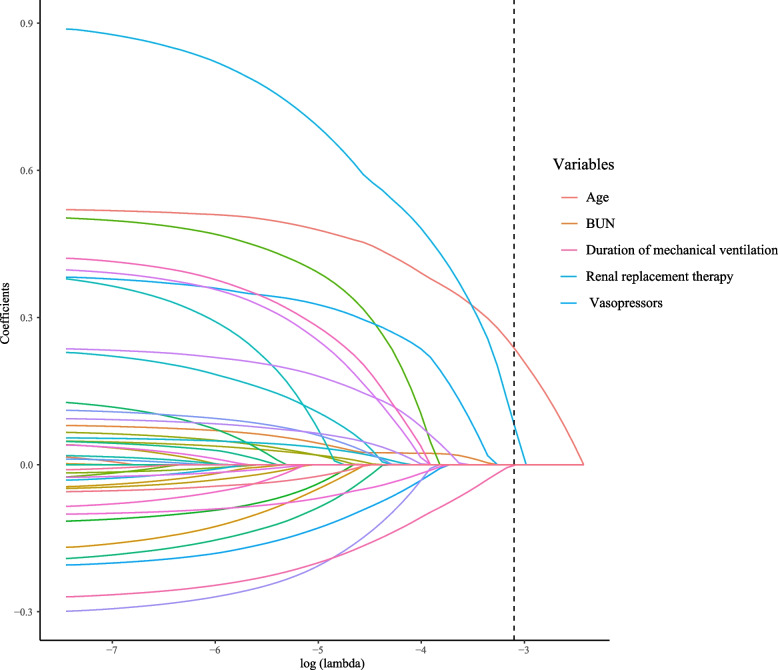
Screening of confounding factors by the least absolute shrinkage and selection operator regression

The result of LASSO regression indicated that age, BUN, vasopressor, RRT and duration of mechanical ventilation might be confounding factors for this study (Fig. 2). Table 2 provides the results regarding the correlation analysis between RDW and 30-day in-hospital mortality. In the univariate COX regression analysis (Table 2), we found that high RDW was a risk factor of 30-day in-hospital mortality of VAP patients (Model 1: HR=1.62, 95%CI: 1.28-2.03, *P*<0.001). After adjusting all confounding factors (Table 2), multivariate COX regression analyses showed that high RDW level was still associated with 30-day in-hospital mortality of VAP patients by using non-high RDW group as the reference (Model 2: HR=1.29, 95%CI: 1.01-1.63, *P*=0.040).

**Table 2:** The association of RDW and 30-day in-hospital mortality in VAP patients by using univariate and multivariate COX regression analyses

Variables	Model 1		Model 2	
	HR (95% CI)	*P*	HR (95% CI)	*P*
RDW				
Non-high level group	Ref		Ref	
High level group	1.62 (1.28-2.03)	<0.001	1.29 (1.01-1.63)	0.040

*VAP* ventilator-associated pneumonia, *RDW* red blood cell distribution width, *HR* hazard ratio, *CI* confidence interval, *Ref* reference

Model 1 did not adjust any variables

Model 2 adjusted for age, blood urea nitrogen, vasopressor, renal replacement therapy and duration of mechanical ventilation.

### Subgroup analyses

The subgroup analyses were performed with 30-day in-hospital mortality as the outcome, RDW level as the independent variable, and non-high RDW level as the reference. Table 3 shows the result of subgroup analyzes based on age, congestive heart failure, sepsis, respiratory failure, RRT and duration of mechanical ventilation. The relationship of high RDW level and 30-day in-hospital mortality was statistically significant for VAP patients who were younger than 60 years (HR=1.77, 95%CI: 1.08-2.92, *P*=0.024), had not a history of congestive heart failure (HR=1.37, 95%CI: 1.00-1.88, *P*=0.049), had the history of sepsis (HR=1.35, 95%CI: 1.04-1.76, *P*=0.026), had not received RRT (HR=1.37, 95%CI: 1.04-1.81, *P*=0.027) and had a duration of mechanical ventilation for more than 7 days (HR=1.75, 95%CI: 1.21-2.54, *P*=0.003).

**Table 3:** Subgroup analyses based on age, congestive heart failure, sepsis, respiratory failure, RRT and duration of mechanical ventilation

Variables	HR (95% CI)	*P*
Age		
<60	1.77 (1.08-2.92)	0.024
≥60	1.20 (0.91-1.58)	0.198
Congestive heart failure		
No	1.37 (1.00-1.88)	0.049
Yes	1.13 (0.78-1.64)	0.511
Sepsis		
No	1.12 (0.62-2.01)	0.716
Yes	1.35 (1.04-1.76)	0.026
Respiratory failure		
No	1.29 (0.87-1.92)	0.211
Yes	1.27 (0.94-1.71)	0.122
RRT		
No	1.37 (1.04-1.81)	0.027
Yes	0.98 (0.61-1.57)	0.924
Duration of mechanical ventilation		
<7 days	1.07 (0.78-1.47)	0.670
≥7 days	1.75 (1.21-2.54)	0.003

*HR* hazard ratio, *CI* confidence interval, *RRT* renal replacement therapy

### Comparisons of RDW and SOFA score and SAPS II score in predicting the 30-day in-hospital mortality for VAP patients

To investigate whether RDW is a good predictor in predicting the 30-day in-hospital mortality for VAP patients, we performed a comparative analysis of RDW and SOFA score and SAPS II score. As shown in Table 4, the area under curve (AUC) value of RDW was 0.705 (95%CI: 0.689-0.721), was higher than SOFA score (AUC=0.589, 95%CI: 0.572-0.606, *P*<0.001) and SAPS II score (AUC=0.646, 95%CI: 0.626-0.663, *P*<0.001), respectively. The result indicated that RDW had a better prognostic value in predicting 30-day in-hospital mortality for VAP patients than SOFA score and SAPS II score.

**Table 4:** Comparisons of RDW and SOFA score and SAPS II score in predicting the 30-day in-hospital mortality for VAP patients

Variables	AUC	95% CI	*P*
RDW level	0.705	0.689-0.721	Ref
SOFA	0.589	0.572-0.606	<0.001
SAPSII	0.646	0.629-0.663	<0.001

*RDW* red blood cell distribution width, *SOFA* sequential organ failure assessment, *SAPS II* simplified acute physiology score II, *VAP* ventilator-associated pneumonia, *AUC* area under curve, *CI* confidence interval, *Ref* reference

## Discussion

In this study, we investigated the correlation between RDW and 30-day in-hospital mortality in VAP patients, and explored predictive value of RDW for VAP patients. Its significant finding was that high RDW level was identified as a risk factor of 30-day in-hospital mortality among VAP patients. Moreover, the RDW exhibited a better prognostic value compared to both SOFA and SAPS II scores in predicting 30-day in-hospital mortality of VAP patients.

RDW has been regarded as an indicator of inflammation, which could be easily obtained by routine blood tests [14]. It is well known that an elevated RDW level may indicate the presence of anemia, iron deficiency, hematopoietic abnormalities, or congenital erythrocyte disorders [15, 16]. Previous studies have suggested that RDW was associated with a variety of diseases, including cardiovascular diseases [8], coronavirus disease 2019 [17], cervical cancer [18], sepsis [19] and critical illness admitted in the ICU [20]. Han YQ, et al. reported that among 36,532 adults with critical illness, an increased RDW level was significantly associated with higher in-hospital and 4-year mortality [20]. Additionally, a study conducted by Zhang L, et al demonstrated that alterations in RDW levels may serve as a mediator for the impact of bronchoscopy on VAP patient prognosis [21]. Nevertheless, the relationship between RDW and VAP remains unclear based on published studies. In the present study cohort, we observed that VAP patients in the death group exhibited higher levels of RDW, which was consistent with previous studies [22, 23]. Importantly, we performed COX regression analyses to investigate the prognostic value of RDW for VAP patients, and observed that high RDW level was a risk factor for 30-day in-hospital mortality in VAP patients. In addition, the relationship was also robust in several populations, such as patients were younger than 60 years, or had not a history of congestive heart failure, or had a history of sepsis, or had not received RRT, or had a duration of mechanical ventilation for more than 7 days. Additionally, we also assessed the relationship of RDW and 30-day in-hospital mortality among non-VAP patients. Our findings indicated that high RDW levels were significantly associated with an increased risk of 30-day in-hospital mortality in non-VAP patients (Supplemental Table 2; HR=1.71, 95% CI: 1.59-1.83; *P*<0.001). Furthermore, we conducted a mediating analysis to investigate the mediator role of VAP in the association of RDW level and 30-day in-hospital mortality. The findings indicated that the mediating effect did not significant (Supplemental Table 3; percentage Mediated= -0.020, 95%CI: -0.069, 0.029; *P*=0.423), suggesting that VAP may not serve as a mediator between RDW and 30-day in-hospital mortality.

A variety of severity scores have been utilized for the prognosis of VAP patients so far [24–26]. Boeck L, et al., showed that SOFA score might be a predictor of VAP [25]. However, we also compared the prognostic value of RDW and severity scores (including SOFA score and SAPS II score), which revealed that RDW exhibited superior predictive power in predicting 30-day in-hospital mortality for VAP patients compared to both SOFA and SAPS II scores. Overall, RDW, as a simple and inexpensive parameter, might be helpful in assessing 30-day in-hospital mortality in VAP patients.

The underlying mechanisms responsible for the association between high RDW levels and 30-day in-hospital mortality among VAP patients remain unknown. Some reports have indicated that RBC homeostasis may be effect in VAP patients, resulting in an elevation of the RDW level [21, 27]. Similarly, inflammation might play a crucial role in the association between RDW and 30-day in-hospital mortality in VAP patients.

Inflammation may impede the maturation process of red blood cells, leading to reticulocytosis and an elevated level of RDW [28, 29]. Further investigation is warranted to elucidate this mechanism.

To our knowledge, this is the first study to show an association between RDW and 30-day in-hospital mortality in VAP patients, providing a reference for the screening and clinical management of prognostic markers for VAP. However, there are some limitations for our study. Firstly, due to the design of the retrospective cohort study, there might be a selection bias in this study. In addition, MIMIC-IV database did not record information on the diseases that led to ventilation, which could introduce further selection bias. A multicenter prospective study is needed to validate our results. Secondly, due to MIMIC-IV database limitations, the RDW data collected in this study only came from measurements taken within the first day (24 h) of patient admission in the ICU. Although mediation analysis suggests that VAP is not a mediating factor between RDW and 30-day in-hospital mortality, we still need to acknowledge this limitation. Future research may require collecting RDW data over a longer period after admission to verify the prognostic value of RDW in 30-day in-hospital mortality of VAP patients.

## Conclusion

RDW may be identified as a significant biomarker for 30-day in-hospital mortality for patients admitted to the ICU, regardless of VAP. In addition, RDW also exhibited a superior predictive value for 30-day in-hospital mortality of VAP patients compared to SOFA and SAPS II scores.

### Supplementary Information

**Additional file 1: Supplemental Table 1.** Sensitivity analysis of missing data before and after interpolation. **Supplemental Table 2.** The association of RDW and 30-day in-hospital mortality in non-VAP patients. **Supplemental Table 3.** The mediating analysis of VAP in the association of RDW level and 30-day in-hospital mortality. 

## Data Availability

The datasets generated and/or analyzed during the current study are available in the MIMIC-IV database, https://mimic.physionet.org/iv/.

## Abbreviations

VAP: Ventilator-associated pneumonia
ICU: Intensive care unit
RDW: Red blood cell distribution width
RBC: Red blood cells
MIMIC-IV: Medical Information Mart for Intensive Care IV
SBP: Systolic blood pressure
DBP: Diastolic blood pressure
WBC: White blood cell count
INR: International normalized ratio
PT: Prothrombin time
INR: International normalized ratio
BUN: Blood urea nitrogen
CCI: Charlson comorbidity index
GCS: Glasgow Coma Scale
SOFA: Sequential organ failure assessment
SAPS II: Simplified acute physiology score II
RRT: Renal replacement therapy
