# Supplementary material for: Relation between red blood cell distribution width and 30-day in-hospital mortality of patients with ventilator-associated pneumonia
Source: BMC Infect Dis. 2023 Oct 18;23:696. doi: 10.1186/s12879-023-08692-0 (PMC10585831; doi:10.1186/s12879-023-08692-0)
Supplement: Supplementary file 1 — Additional file 1: Supplemental Table 1. Sensitivity analysis of missing data before and after interpolation. Supplemental Table 2. The association of RDW and 30-day in-hospital mortality in non-VAP patients. Supplemental Table 3. The mediating analysis of VAP in the association of RDW level and 30-day in-hospital mortality. [file 12879_2023_8692_MOESM1_ESM.docx]

**Supplemental Table 1. Sensitivity analysis of missing data before and after interpolation**

| Variables | Before the interpolation | After the interpolation | *P* |
| --- | --- | --- | --- |
| Weight, kg, Mean ± SD | 86.13 ± 27.44 | 85.16 ± 26.67 | 0.344 |
| Marital status, n (%) |  |  | 0.983 |
| Divorced | 111 (8.31) | 123 (7.97) |  |
| Married | 651 (48.76) | 758 (49.13) |  |
| Single | 440 (32.96) | 505 (32.73) |  |
| Widowed | 133 (9.96) | 157 (10.17) |  |
| SBP, mmHg, Mean ± SD | 124.71 ± 26.60 | 124.70 ± 26.60 | 0.993 |
| DBP, mmHg, Mean ± SD | 69.08 ± 18.49 | 69.08 ± 18.49 | 0.999 |
| Heart rate, bpm, Mean ± SD | 90.73 ± 21.46 | 90.71 ± 21.48 | 0.970 |
| Respiratory rate, bpm, Mean ± SD | 19.95 ± 6.19 | 19.88 ± 6.12 | 0.761 |
| Temperature, ℃, Mean ± SD | 36.76 ± 1.02 | 36.74 ± 1.01 | 0.611 |
| SPO2, %, Mean±SD | 96.92 ± 4.56 | 96.92 ± 4.56 | 0.998 |
| GCS, score, Mean ± SD | 8.00 (4.00, 12.00) | 8.00 (4.00, 12.00) | 1.000 |
| WBC, K/uL, M (Q_1_, Q_3_) | 11.90 (8.70, 16.30) | 11.90 (8.70, 16.30) | 1.000 |
| PLT, K/uL, M (Q_1_, Q_3_) | 193.00 (135.00, 254.00) | 193.00 (135.00, 254.00) | 1.000 |
| Hemoglobin, g/dL, Mean ± SD | 10.90 ± 2.43 | 10.90 ± 2.43 | 1.000 |
| Creatinine, mg/dL, M (Q_1_, Q_3_) | 1.00 (0.80, 1.60) | 1.00 (0.80, 1.60) | 0.981 |
| INR, M (Q_1_, Q_3_) | 1.30 (1.10, 1.50) | 1.20 (1.10, 1.50) | 0.083 |
| BUN, mg/dL, M (Q_1_, Q_3_) | 21.00 (14.00, 34.00) | 21.00 (14.00, 34.00) | 0.983 |
| Glucose, mg/dL, M (Q_1_, Q_3_) | 139.00 (112.00, 181.50) | 139.00 (111.00, 181.00) | 0.971 |
| Anion gap, mEq/L, Mean ± SD | 15.43 ± 4.83 | 15.43 ± 4.82 | 0.997 |

SBP, systolic blood pressure; DBP, diastolic blood pressure; SpO2, pulse oxygen saturation; GCS, Glasgow Coma Scale; WBC, white blood cell count; PLT, platelet count; INR, international normalized ratio; BUN, blood urea nitrogen; SD, standard deviation.

**Supplemental Table 2 The association of RDW and 30-day in-hospital mortality in non-VAP patients**

| Variables | Multivariate COX regression analysis * | |
| --- | --- | --- |
|  | HR (95% CI) | *P* |
| RDW level |  |  |
| Non-high level group | Ref |  |
| High level group | 1.71 (1.59-1.83) | <0.001 |

VAP, ventilator-associated pneumonia; RDW, red blood cell distribution width; HR, hazard ratio; CI, confidence interval; Ref, reference

*adjusted for age, gender, ethnicity, weight, diastolic blood pressure, heart rate, respiratory rate, temperature, creatinine, international normalized ratio, blood urea nitrogen, glucose, pulse oxygen saturation, sepsis, respiratory failure, vasopressor, renal replacement therapy, vancomycin, penicillin, aminoglycoside, thiamine, streptococcus pneumoniae, proteus, and duration of mechanical ventilation.

**Supplemental Table 3 The mediating analysis of VAP in the association of RDW level and 30-day in-hospital mortality**

| Effect | Odds ratio or excess relative risk or percentage | 95% CI | *P* |
| --- | --- | --- | --- |
| Odds ratio total effect | 1.780 | 1.645 to 1.915 | <0.0001 |
| Odds ratio CDE | 1.780 | 1.645 to 1.915 | <0.0001 |
| Odds ratio NDE | 1.780 | 1.645 to 1.915 | <0.0001 |
| Odds ratio NIE | 1.000 | 0.9997 to 1.0001 | 0.4228 |
| Total excess relative risk | 0.780 | 0.645 to 0.915 | <0.0001 |
| Excess relative risk due to CDE | 0.781 | 0.646 to 0.916 | <0.0001 |
| Excess relative risk due to NDE | 0.780 | 0.645 to 0.915 | <0.0001 |
| Excess relative risk due to NIE | -0.0002 | -0.0005 to 0.0002 | 0.423 |
| Percentage mediated | -0.020 | -0.069 to 0.029 | 0.4233 |
| Percentage due to interaction | -0.112 | -0.327 to 0.103 | 0.3071 |
| Percentage eliminated | -0.123 | -0.361 to 0.114 | 0.3095 |

CDE, controlled direct effect; NDE, natural direct effect; NIE, natural indirect effect; CI, confidence interval; VAP, ventilator-associated pneumonia; RDW, red blood cell distribution width.
